# Neurosyphilis Presenting as Asymptomatic Optic Perineuritis

**DOI:** 10.1155/2012/621872

**Published:** 2012-02-26

**Authors:** Sarah E. Parker, John H. Pula

**Affiliations:** Neuro-ophthalmology Unit, University of Illinois College of Medicine at Peoria, 530 NE Glen Oak Avenue, Peoria, IL 61611, USA

## Abstract

*Introduction*. Syphilis is a sexually transmitted disease that is known as “the great imitator” due to its wide variety of clinical presentations, including ocular disorders. There has been an increase in the rate of syphilis in the United States, especially in persons with HIV. We report a case of optic perineuritis in an asymptomatic male secondary to central nervous system (CNS) syphilis. *Case Report*. A 41-year-old man was found to have bilateral disc edema on a routine exam. Brain MRI was unremarkable, and lumbar puncture revealed a normal opening pressure, with an elevated cerebrospinal fluid white cell count. Orbit MRI showed optic nerve sheath expansion and enhancement, consistent with optic perineuritis. He tested positive for syphilis based on serum RPR and FTA-ABS. *Conclusion*. Ophthalmologic findings, including disc edema, may be the presenting features of CNS syphilis. Even in asymptomatic persons, perineuritis should be considered early, as diagnosis and treatment are imperative given the progressive nature of the disease.

## 1. Introduction

Syphilis is a sexually transmitted disease caused by the spirochete *treponema pallidum*. Since the first known outbreak in Europe in 1494 [1], the disease has collected many nicknames. This “great imitator” appears on myriad differential diagnoses due to it is varied presentations, especially in neurosyphilis. It was known as “The Great Pox” and the “disease of the century” [1]. After the discovery of penicillin to treat syphilis, the disease became less common and to an entire generation of physiciansm “the great imitator” became a diagnosis widely considered but rarely diagnosed. Increasingly, the name “the great imitator” has been used to refer to other diseases such as HIV. However, there has also been a recent increase in the rates of syphilis, especially in the HIV population [2].

The manifestations of neurosyphilis were recognized in 1822 by Antoine-Laurent Bayle [1]. The most notable is tabes dorsalis, known as general paresis of the insane, dementia paralytica, or progressive paralysis. However, ocular findings are also seen in neurosyphilis and can be the presenting feature.

## 2. Case Report

A 41-year-old male was referred for blurred disk margins and possible papilledema from his optometrist. He was visually asymptomatic. He had been seen in a hematology-oncology clinic one month prior for swollen groin lymph nodes that were noted to be reactive, but of unknown etiology. He had not had any previous STDs, genital lesions, penile discharge, or rash.

**Figure 1:**
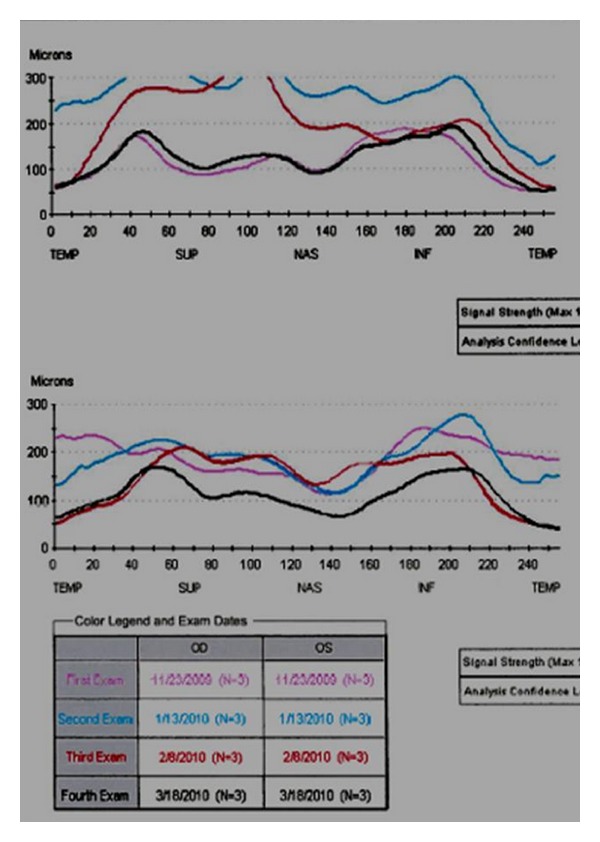
Composite retinal nerve fiber layer OCT. Optical coherence tomography showing extent of disc swelling during clinical course. At presentation (magenta line), disc edema is mild in the right eye, but more severe in the left eye. One and three weeks later (red and blue lines), disc edema has increased to both eyes. After treatment with antibiotics (black line), disc edema has essentially resolved.

On exam, visual acuity was 20/25+ OD and 20/20− OS. Ishihara color plates were 11/11 OU. He had a trace afferent pupillary defect OS. Intraocular pressures were 12 OD and 18 OS. He had asymmetric disc edema, greater in the left eye. Optical coherence tomography showed a retinal nerve fiber layer (RNFL) average thickness of 188 microns OS with concentric circumferential elevation and an average RNFL thickness of 118 OD with inferonasal elevation (Figure 1).

**Figure 2:**
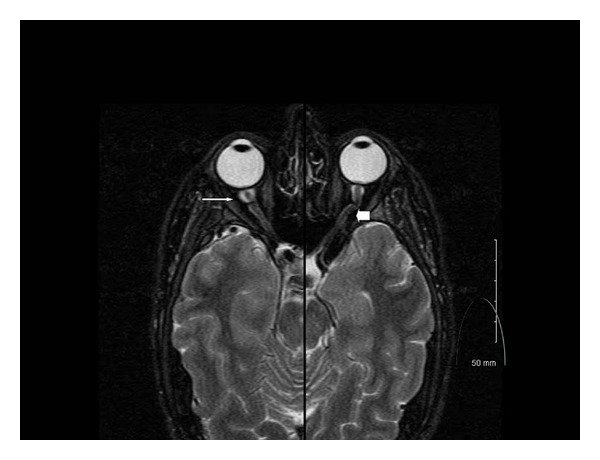
MRI of optic nerves. Merged axial T2 MRI at the level of the optic nerves shows significantly increased optic nerve sheath expansion near the globe (thin arrow), which extends through the course of the optic nerve sheath (thick arrow).

**Figure 3:**
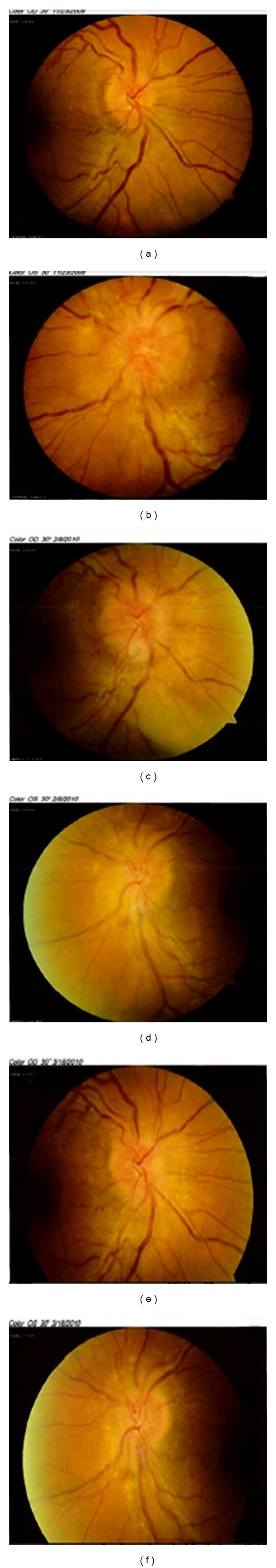
Composite of fundus appearances over time. Fundus photos showing extent of disc edema over time: at presentation (a, b), pretreatment with highest-grade edema (c, d), and posttreatment (e, f).

**Figure 4:**
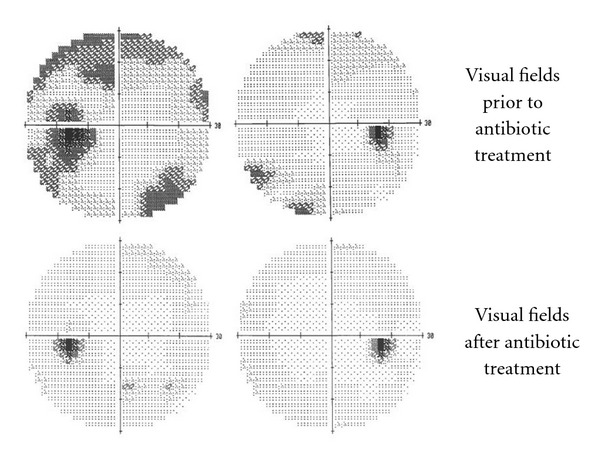
Visual field examination before and after treatment. Humphrey visual field testing shows improvement and resolution of enlarged blind spot after antibiotic treatment.

Brain MRI and MRV with and without contrast were unremarkable, without mass lesions or venous occlusion. He underwent a lumbar puncture which showed a normal opening pressure of 18 cm H_2_O, normal protein, and glucose, and 72 white cells/mm^3^, predominately lymphocytes. Within 2 weeks of presentation, he developed headaches and eye pain, especially when bending over. Repeat examination showed worsening disc edema in the right eye and enlargement of the blind spot on visual field testing. Because his presentation was not consistent with papilledema, orbit MRI was performed, which showed increased optic nerve sheath fluid, especially posterior to the globes (Figure 2) and contrast enhancement of the optic nerve sheaths. Since the clinical and imaging findings appeared consistent with optic perineuritis, he was tested for secondary causes. He tested positive for syphilis with a rapid plasma reagent (RPR), with a titer of 1 : 128. FTA-ABS measured for confirmation was also positive. HIV testing was negative. He was placed on IV Penicillin 3,000,000 Units every 4 hours for 14 days. Repeat testing two weeks after antibiotic treatment showed marked improvement in both his symptoms and the extent of disc edema (Figure 3). Visual field testing also improved after antibiotics, with resolution of the enlarged blind spot (Figure 4).

## 3. Discussion

Syphilis is a sexually transmitted disease that consists of three stages. The primary stage is a painless chancre that occurs after a 3–6-week incubation period and usually heals within six weeks. The secondary stage occurs after 1-2 months and is characterized by myriad symptoms including a macular rash on the palms and soles of the feet, fever, body aches, arthralgias, and malaise. Two thirds of patients with untreated secondary syphilis remain asymptomatic after this stage while the others develop tertiary syphilis, which can occur months to years after the initial infection. Neurosyphilis can occur at any stage and is defined as infiltration of *T. pallidum* into the central nervous system [3].

The Centers of Disease Control defined neurosyphilis as any syphilis stage with a reactive CSF VDRL [3]. Presumptive neurosyphilis is defined as (1) any stage of syphilis, with (2) a nonreactive CSF VDRL, (3) increased CSF protein or white blood cell (WBC) count in the absence of other known causes of these abnormalities, and (4) clinical symptoms or signs consistent with neurosyphilis without other known causes for these clinical abnormalities [3].

Ocular findings described in neurosyphilis include retinitis, retinal vasoocclusion, iritis, papillitis, keratic precipitates, viritis, periphlebitis, serous and exudative retinal detachments, optic neuritis, and perineuritis [2–4]. The ocular motor nerves can also be affected by neurosyphilis [3]. The most common ocular finding in tertiary syphilis is uveitis which occurs in 2–5% of patients [2]. In our patient, the initial presentation of asymptomatic bilateral disc edema would usually suggest papilledema. However, the normal CSF pressure and elevated CSF WBC count, along with the orbit MRI findings, suggested perineuritis.

Optic perineuritis is a relatively uncommon inflammatory condition which may be mistaken for optic neuritis or optic nerve sheath meningioma. The condition is often idiopathic. Typical clinical features include eye pain, mildly decreased visual acuity, and a variety of different visual field defects. On exam, not all persons with optic perineuritis have disc edema, but, when present, edema may be bilateral. Optic nerve sheath enhancement, often with enhancement of the orbital fat is a typical MRI finding. Unlike optic neuritis, the optic nerve usually does not show prominent gadolinium enhancement [5].

The incidence of syphilis is increasing in some groups such as the HIV population. Given that syphilis is a progressive disease that can have a serious impact on the central nervous system and the eyes, it is important to make the diagnosis early so that treatment can be initiated. We believe our report provides the first example of an initially asymptomatic patient presenting with bilateral optic perineuritis as a first manifestation of neurosyphilis.
